# Automated Anxiety Detection System Integrating a Brain–Computer Interface for Neurofeedback Applications

**DOI:** 10.3390/s26134004

**Published:** 2026-06-24

**Authors:** Mashael Aldayel, Abeer Al-Nafjan

**Affiliations:** 1 Information Technology Department, College of Computer and Information Sciences, King Saud University, Riyadh 11543, Saudi Arabia; maldayel@ksu.edu.sa; 2 Computer Science Department, College of Computer and Information Sciences, Imam Mohammad Ibn Saud Islamic University (IMSIU), Riyadh 11432, Saudi Arabia

**Keywords:** brain–computer interface, electroencephalography, neurofeedback, anxiety, deep learning

## Abstract

Anxiety disorders pose an increasing challenge to the mental health of individuals, particularly in regions with limited healthcare access. This study investigated the potential of integrating a brain–computer interface for processing electroencephalography (EEG) data with deep learning models to accurately classify anxious and non-anxious states. In the first phase, a convolutional neural network (CNN) was developed and validated on the public GAMEEMO dataset, achieving a classification accuracy of 95.72%. In the second phase, we conducted a separate experimental validation with seven participants (aged 18–60 years) using a within-subjects design. The protocol comprised a custom Stroop test to elicit acute cognitive stress and anxiety-related arousal, followed by a guided 4–7–8 breathing exercise to induce relaxation. EEG data from this experiment were used to classify anxious versus non-anxious states with the same CNN architecture after domain adaptation. On this self-collected dataset, the CNN achieved an accuracy of 86.58%. These results demonstrate proof-of-concept transferability while highlighting the performance gap between controlled benchmark data and real-world, small-sample recordings. The deep learning model can subsequently be coupled with neurofeedback techniques to manage anxiety levels. Overall, the findings support the potential of the developed automated system for detecting stress-induced anxious states, with possible future integration into neurofeedback-based management systems.

## 1. Introduction

Anxiety disorders are mental health problems that can result in physical consequences. Unfortunately, there is growing evidence that excessive anxiety can dramatically weaken the immune system. Anxiety is essentially long-term stress that causes the body to release large quantities of stress hormones, which degrade the body’s functions. Anxiety can also affect academic performance, including memory skills, which can increase the difficulty of learning and recycling information [1].

Detecting anxiety is essential for effective management and intervention. Recent technological advances have resulted in new ways to detect and manage anxiety [2]. For example, wearable sensors can track heart rate, galvanic skin response, and other biomarkers of stress and anxiety levels [3]. Mobile applications connected to handheld or standalone devices can provide additional assistance such as real-time feedback and guidance through anxiety management techniques [3]. Virtual reality (VR) is being explored as a way to simulate anxiety-inducing environments (e.g., public speaking or flying) so that users can face their fears in a controlled environment [4].

Brain–computer interfaces (BCIs) record brain activity that is then processed by a computer, which allows individuals to monitor their own brain activity in real time [5]. BCI applications include assistive modules for severely paralyzed patients to help them control external devices or communicate, as well as neurofeedback (NF) to self-regulate brain activity for treating neurological and psychological disorders such as epilepsy, attention-deficit hyperactivity disorder, and anxiety, as well as to improve cognitive performance in healthy individuals [6]. NF systems rely on accurate and reliable detection of the user’s mental state as a prerequisite for delivering meaningful real-time feedback. Specific applications are designed for each condition, and some may work for multiple disorders at the same time [7].

The motivation for this research came from a desire to address the increasing prevalence of anxiety disorders and their detrimental effects on individuals’ quality of life. Emerging technologies such as BCI and deep learning (DL) offer a promising pathway toward innovative and accessible anxiety management solutions that could empower individuals to take responsibility for their mental health and improve their well-being and productivity.

A critical first step toward such systems is the reliable automated detection of anxiety-related states from EEG signals. This paper therefore focuses on the development of an EEG-based system for detecting stress-induced anxious states using BCI and DL—a foundation on which real-time NF interventions can subsequently be built. Accurately classifying emotional states such as anxiety from complex brain signals requires the integration of advanced signal processing and machine-learning (ML) techniques.

The remainder of this paper is structured as follows. Section 2 reviews related work. Section 3 presents the proposed automated anxiety detection system. Section 4 presents the experimental design, while Section 5 discusses the results. Section 6 concludes the paper.

## 2. Literature Review

This section surveys prior work on EEG-based detection of affective states and on closed-loop interventions relevant to anxiety modulation. We first summarize experimental paradigms, datasets, and neurofeedback applications, then outline common preprocessing, feature extraction, and classification pipelines used in the literature. This context motivates our design choices and highlights the remaining gaps our study addresses.

Aldayel et al. [8] explored various ML techniques for improving the accuracy of anxiety detection using EEG data by comparing feature extraction methods, such as discrete wavelet transform (DWT) and power spectral density (PSD), with labeling techniques such as the Hamilton anxiety rating scale and self-assessment manikin (SAM). They demonstrated that combining a random forest (RF) with DWT features resulted in the highest classification accuracy of 87.5%. Chen et al. [9] designed a closed-loop NF experiment in which subjects were trained to regulate their emotional states by receiving real-time feedback from their EEG signals. Their results showed that a support vector machine (SVM) could classify anxiety and non-anxiety states with an accuracy of over 90%. Al-Nafjan et al. [10] extracted features such as PSD and frontal asymmetry from the Database for Emotion Analysis Using Physiological and Audiovisual Signals (DEAP) dataset to train a deep neural network (DNN) to classify human emotions from EEG signals, and showed that it outperformed traditional classifiers such as RF in terms of classification accuracy. Their results highlighted the potential of DL techniques for detecting emotions from EEG signals, especially when large datasets are available.

Birch et al. [11] studied the effects of home-based NF on anxiety levels in participants with chronic pain. Prior to training, many of the participants exhibited clinical anxiety, but post-intervention, there was a notable reduction in anxiety, with many participants achieving normal levels. The statistically significant improvement in anxiety during follow-up assessments demonstrates the potential effectiveness of NF at alleviating anxiety associated with chronic pain. Kamińska et al. [4] studied the use of EEG signals and VR to induce and measure stress and relaxation in participants. They employed specific experimental protocols including the Stroop test to induce stress and delved into the technical aspects of EEG signal processing and analysis, including the use of specific frequency bands and signal filtering techniques.

Jeunet et al. [12] optimized NF and BCI training by focusing on personalized mental strategies and non-task-specific approaches. They emphasized the importance of guiding users toward more effective mental strategies such as kinesthetic motor imagery over visual imagery to enhance the BCI performance. Mindfulness meditation was found to reduce anxiety while improving attentional control. Arpaia et al. [13] investigated the effects of NF training on anxiety, depression, and self-esteem levels. They employed the Wilcoxon signed-rank test to analyze pre- and post-training scores in various scales designed to measure anxiety and depression, which revealed no statistically significant differences. However, a notable reduction in the self-reported depression scores was observed, which suggests that NF may help with emotional regulation. They also tried to correlate physiological changes with emotional states by focusing on the high-beta power in midline locations of EEG signals, and found that a decrease in the high-beta power during training sessions was associated with improved emotional regulation capacity. However, statistical significance was not achieved, which highlighted the need for larger sample sizes and extended NF sessions to draw more definitive conclusions.

Huang et al. [14] developed a real-time EEG-based BCI neurofeedback system to help participants regulate their emotions. The system was trained with 20 adults over 10 sessions to modulate positive, neutral, and negative states using real-time EEG decoding based on wideband features and an SVM classifier. Across sessions, both classification accuracy and spectral topographies improved, with emotion-specific increases in beta/gamma power over temporal and frontal regions. These experimental results showed that participants progressively enhanced their ability to regulate emotional states through BCI-based neurofeedback training.

Tan et al. [15] conducted an open-label randomized trial of a home-based, mindfulness-informed BCI game for young adults with moderate anxiety, using a Muse EEG headband and an individualized SVM classifier to provide real-time arousal feedback. Across eight 30 min sessions over two weeks, participants showed significant pre–post reductions in anxiety, depression, stress, and insomnia, plus improved mindfulness and emotion regulation scores, supported by increased frontal alpha and reduced high-beta power in EEG. Antle et al. [16] proposed Mind-Full, a low-cost, EEG-based neurofeedback game designed to teach anxiety and attention self-regulation to children living in poverty in Nepal. Using a NeuroSky headset and familiar, culturally grounded game scenarios, children aged 5–11 received real-time feedback on relaxation (alpha/theta) and attention (beta), with outcomes assessed for feasibility, transfer to classroom behavior, and maintenance over time via a waitlist control design. The study showed that children could reliably use the system and sustain some gains in self-regulation, suggesting that portable, game-based neurofeedback can support emotional and attentional regulation in low-resource school settings.

The studies summarized below span laboratory tasks (e.g., Stroop, VR scenarios), clinical or home-based interventions, and public datasets commonly used for affective computing. Table 1 organizes these works by stimuli, participants, EEG setup, and targeted affective states to provide a comparable overview.

Having outlined the experimental settings and affective targets, we next synthesize the computational pipelines used across studies. Table 2 synthesizes representative studies by their preprocessing steps, feature sets, and classifiers, and notes recurring limitations.

BCI devices range from consumer-grade systems like Emotiv EPOC [1,17] to research-grade devices like NuAmps Amplifier [13]. Innovative combinations like EEG headsets with tablets [16] have real-world applicability. The computational techniques used in related works can be categorized according to the following:

- Preprocessing methods including artifact removal [4,9] and bandpass filtering [9,14] are often employed to ensure signal quality. Normalization [18] further improves reliability across datasets.
- Feature extraction in the reviewed studies commonly relies on frequency-domain signal measures such as PSD [9,10,15] and band-specific power values. These measures are typically computed using signal-processing methods such as Fourier transform (FFT) or short-time Fourier transform (STFT) [14,15]. Advanced techniques such as Hjorth features and fractal dimensions [1] cater to specific tasks while balancing efficiency and adaptability. Frequency-domain features such as band power across EEG bands [1,10,14] and task-specific features like frontal asymmetry are commonly extracted because of their usefulness for capturing brain activity and tailoring applications.
- Classification algorithms include SVM, the most widely used model [1,9,14], which can be attributed to its ability to handle high-dimensional data. Advanced approaches including hybrid models like the stacked sparse autoencoder (SSAE) [1] and convolutional neural network (CNN) [4] have shown growing sophistication. However, ongoing limitations include small sample sizes [11,15], device constraints [17], and data imbalances [1].

## 3. Proposed System

**Figure 1:**
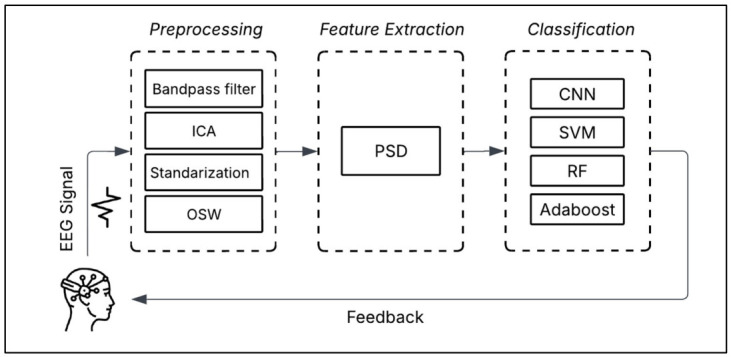
Framework of the automated anxiety detection system.

This section presents the end-to-end pipeline for detecting stress-induced anxious states from EEG signals. The approach follows the conventional BCI processing flow, including signal acquisition, preprocessing, feature extraction, and classification. Figure 1 illustrates the overall framework of the proposed system.

EEG signals are captured by an Emotiv Epoch+ headset. In the preprocessing stage, noise and artifacts such as movement and environmental interference are filtered out of the raw signals via a bandpass filter, independent component analysis (ICA), and standardization. In the feature extraction stage, relevant features are identified and selected by techniques such as the overlap sliding window (OSW) and PSD. The extracted features are subsequently fed into the classification stage, where ML algorithms (e.g., RF, CNN, SVM) are applied to classify the user’s state as anxious or non-anxious.

Based on the outcome of the classification stage, the system can provide EEG-guided behavioral feedback, such as guided breathing exercises or CBT-based prompts, to support stress and anxiety management. This should be distinguished from classical neurofeedback, which requires continuous real-time presentation of the user’s own EEG activity. In the current study, the implemented contribution is the EEG-based anxiety detection module, which may serve as a foundation for future neurofeedback applications. This feedback loop allows the proposed system to adaptively help the user regulate their anxiety in real-time.

### 3.1. Emotional States

**Figure 2:**
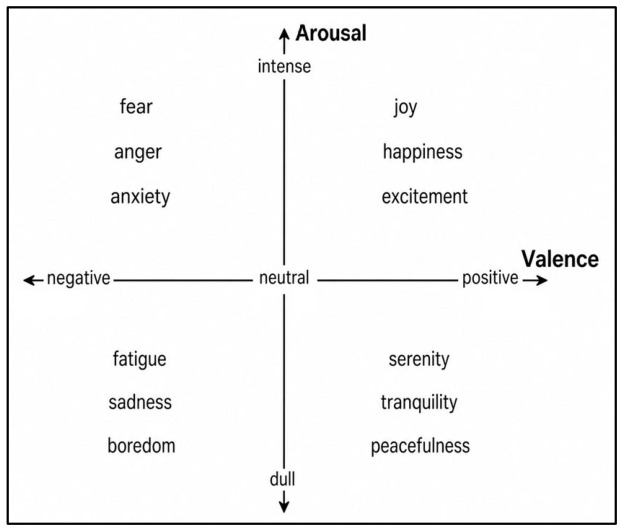
Valence–arousal circumplex model for representing emotional states.

We ground our labeling strategy in the valence–arousal circumplex to align EEG-derived features with self-reported affect. As shown in Figure 2, the valence–arousal circumplex model was utilized to visualize emotional states based on their intensity (i.e., arousal) and pleasantness (i.e., valence). The valence ranges from negative emotions such as anxiety to positive emotions such as happiness. The arousal ranges from low-arousal states such as calmness to high-arousal states such as anxiety [19]. Thus, emotional states are distributed in the following quadrants:

- High-arousal, negative valence (HANV; e.g., anxiety, fear, anger);
- High-arousal, positive valence (HAPV; e.g., joy, happiness, excitement);
- Low-arousal, negative valence (LANV; e.g., boredom, sadness, fatigue);
- Low-arousal, positive valence (LAPV; e.g., serenity, tranquility, peacefulness).

The self-assessment manikin (SAM) measures a person’s emotional response along three dimensions: valence, arousal, and dominance. It provides an intuitive method for subjects to self-report their feelings and bypasses the need for extensive verbal explanations. SAM has been particularly effective in studies involving emotion recognition and for correlating EEG data with emotional states [20,21].

For each sample in our dataset, we define an anxiety label as follows: if valence < 5 (negative emotion) AND arousal > 5 (high intensity), the sample is labeled as “anxious”. Otherwise, it is labeled as “non-anxious”. This mapping is directly based on established affective research [20,21], which characterizes anxiety as a state of high arousal combined with negative valence (unpleasant, worried, tense). The neutral midpoint of 5 on the 9-point Likert scale was excluded from both criteria, ensuring that only clearly negative valence (scores 1–4) and clearly high arousal (scores 6–9) contribute to the “anxiety” label.

### 3.2. Dataset

We selected the GAMEEMO dataset to develop and benchmark our model. As presented in Table 3, the GAMEEMO dataset comprises EEG recordings from 28 participants who played four distinct computer games categorized with the emotional labels of boring, calm, scary, and funny. Participants completed the games in a dark and quiet environment with standardized settings to ensure high graphical quality and immersive auditory and visual stimuli. EEG data were acquired by an Emotiv EPOC+ headset with a 14-channel configuration based on the International 10–20 system. Alakus et al. [20] collected 112 recording files from 28 participants engaged in four distinct games, which produced 38,252 samples. The authors made the dataset publicly available in both raw and preprocessed forms.

In the present study, EEG data went through a cleaning pipeline to ensure high-quality signal fidelity and noise reduction. The GAMEEMO dataset included valence and arousal ratings derived from SAM, which were provided by participants after gameplay. The data were subsequently reformatted from structured Portable Document Format files to the Comma Separated Values format to facilitate enhanced analysis and labeling. After signal segmentation, each EEG segment was labeled based on the valence and arousal ratings provided by the subjects. These ratings were mapped into two emotional states for binary classification: anxious and non-anxious. Segments with a valence less than 5 and arousal greater than 5 were labeled as “anxious” [22]. These scores were directly linked to the segmented EEG data to ensure accurate and consistent labeling for supervised learning.

### 3.3. Preprocessing

To ensure signal quality and comparability with prior EEG emotion studies, we applied established artifact rejection and filtering steps before segmentation and scaling.

EEG data typically contain artifacts such as eye movement, electromyography, and electrocardiogram signals that need to be removed [23]. Research has highlighted that artifact removal and bandpass filtering are essential preprocessing steps for ensuring EEG signal quality [24,25]. In this study, ICA was employed owing to its efficacy at isolating and removing these artifacts, including ocular and muscle artifacts [9,26]. Following artifact removal, a bandpass filter with a range of 1–45 Hz was utilized to capture brain waves associated with different mental states (e.g., delta, theta, alpha, beta, and gamma) while eliminating low-frequency artifacts and high-frequency noise [9,14].

To ensure the dataset was prepared for effective analysis and modeling, we applied data cleaning and transformation steps. During the initial data inspection, we found that a small number of feature columns contained only NaN (missing) values, typically because a specific EEG channel had a complete signal dropout during recording. These columns contained no valid data for any row, so they provided no useful information to the model. Hence, we removed those columns entirely.

To ensure features were on a similar scale and optimize the ML algorithm performance, standardization was employed, which involved transforming the data to have a mean of 0 and standard deviation of 1. Apicella et al. [27] studied the impact of data normalization on domain adaptation on EEG datasets and demonstrated that normalization enhanced the classification performance in EEG-related tasks. The OSW method was employed to segment EEG signals into smaller, meaningful, and manageable data units while preserving temporal continuity. This technique divides continuous time-series EEG data into overlapping segments to enhance the dataset size and ensure sufficient data representation for analysis and classification The OSW method has been demonstrated to be effective at enhancing the robustness of ML models by increasing the quantity of data without introducing redundancy [22].

In the GAMEEMO dataset, each subject-game file contained 38,252 time samples per channel (14 channels, 298.8 s duration at a 128 Hz sampling rate).

### 3.4. Feature Extraction

After OSW, the EEG signals were divided into windows of 4 s (512 samples) with a step size of 1 s (128 samples), generating 99 overlapping windows per file. Across all 112 files, this resulted in a total of 11,088 windows (112 × 99). For each window (epoch), we applied the multitaper method to compute PSD across five frequency bands: delta (1–4 Hz), theta (4–8 Hz), alpha (8–13 Hz), beta (13–30 Hz), and gamma (30–45 Hz). The PSD computation yielded 257 frequency bins per channel, giving a feature vector of 3598 dimensions per window (14 channels × 257 bins).

### 3.5. Class Imbalance

Emotion recognition datasets often exhibit class imbalance, where minority classes such as “anxious” are typically underrepresented compared to majority classes such as “non-anxious”. This imbalance can lead to the training of biased models that favor majority classes and underperform on minority classes.

This imbalance can bias machine learning models toward the majority class, leading to poor performance on minority classes. In our original binary class distribution, the “non-anxious” class contained 9306 samples while the “anxious” class contained only 1782 samples, meaning that anxious instances accounted for approximately 16% of the dataset. We then performed hold-out validation to split the data (80/20), resulting in a training set of 8870 samples (80% of the total 11,088 epochs) with 7444 non-anxious and 1426 anxious instances, and a test set of 2218 samples (20% of the total) that retained the original imbalanced distribution. No synthetic oversampling was applied to the test set.

The split was performed on a subject-wise basis, ensuring that all windows from the same subject were assigned exclusively to either the training or test set, not both.

Recent studies have underscored the critical role of the synthetic minority oversampling technique (SMOTE) for mitigating bias within ML models. SMOTE not only effectively balances datasets but also maintains the overall accuracy, which is indispensable for addressing class imbalance challenges and developing fair and reliable decision-support systems [28]. Borderline-SMOTE is a data augmentation method that is tailored to handle class imbalance by focusing on the borderline samples of the minority class (e.g., anxious). While traditional SMOTE can lead to class overlap by generating synthetic data without considering the distribution of adjacent majority-class samples, Borderline-SMOTE strengthens the minority-class samples near the decision boundary where misclassification is more likely [22]. This targeted oversampling reduces the risk of overlapping classes and improves model generalization [29].

Given the strong class imbalance in our original dataset (9306 non-anxious vs. 1782 anxious), we applied Borderline-SMOTE exclusively to the training set, generating 6018 synthetic samples for the minority class to achieve a balanced training set of 7444 samples per class.

### 3.6. Classification Algorithms

After features are extracted from EEG data, the next step is to train a suitable classifier to differentiate between emotional states. In this study, several classifiers were applied to analyze the EEG data: SVM, RF, AdaBoost, and CNN. SVM is often chosen for EEG analysis because of its ability to handle high-dimensional data, identify optimal decision boundaries, and remain robust against overfitting, particularly in scenarios involving the binary classification of emotional states [30]. Data are plotted in a high-dimensional space to identify a hyperplane that separates classes with the widest possible margin [31].

RF is a supervised ensemble learning algorithm that performs classification and regression tasks by creating a collection of decision trees. Each tree is trained on a random subset of the data, and their outputs are aggregated through majority voting or averaging depending on the problem type. This approach minimizes overfitting while maintaining high accuracy on a wide range of datasets [31].

AdaBoost (Adaptive Boosting) is an ensemble method that combines multiple weak classifiers into a strong classifier. It does not assume feature independence. It iteratively adjusts sample weights, increasing the importance of misclassified instances so that subsequent weak classifiers focus on harder examples. The final prediction is a weighted majority vote, where classifiers with lower error receive higher influence. This approach reduces both bias and variance [32].

CNN is extensively used for EEG-based emotion recognition because of its proficiency in capturing spatial and temporal patterns within EEG signals. Ozdemir et al. [33] employed the CNN architecture to distinguish emotional states by first converting EEG signals into multispectral topology images.

In this study, SVM was implemented by using LinearSVC from scikit-learn 1.6.1. RF was implemented by using RandomForestClassifier from scikit-learn. AdaBoost was implemented by using AdaBoostClassifier with DecisionTreeClassifier as the base estimator. The base decision tree was constrained to a maximum depth of 1 (stumps). CNN was implemented by using the TensorFlow 2.20.0, Keras 3.13.2 library. It featured 1D convolutional layers with 64 and 128 filters, a kernel size of 3, and a rectifier linear unit as the activation function. Max pooling layers were used to reduce the dimensions, and fully connected dense layers including a dropout rate of 0.5 were added to prevent overfitting. The final layer used softmax activation to output class probabilities for anxious and non-anxious states.

### 3.7. Results and Discussion

This section reports the experimental results and their interpretation. To enable a realistic evaluation and to prevent data leakage, we adopted an 80/20 subject-independent hold-out split, where all windows from any given subject were assigned exclusively to either the training set or the test set—never to both. The training set was balanced using Borderline-SMOTE, while the test set retained the original imbalance to reflect real-world conditions. In addition, we performed 5-fold subject-independent cross-validation using GroupKFold with subject identifiers as the grouping parameter, ensuring that all samples from a given subject were kept entirely within one fold. This prevents any overlap of windows from the same subject across training and validation folds, eliminating data leakage.

The following subsections cover: hold-out performance, cross-validation with statistical testing, computational efficiency, feature importance analysis, and a comparison with prior work on the same dataset.

#### 3.7.1. Hold-Out Performance

As shown in Table 4, classifier performance was assessed using standard metrics: accuracy, precision, recall, and F1 score. Similar research has also adopted these standard evaluation metrics [34,35]. Among the traditional machine learning models, RF achieved the highest accuracy (95.13%) and a macro F1 score of 90.51%. Its performance on the minority (anxious) class was reasonable (macro recall = 78.93%, macro F1 = 83.88%), but it was clearly outperformed by the CNN. SVM showed a lower accuracy (89.27%) and a macro F1 of 81.22%; moreover, the SVM used a linear kernel, which might not be optimal for this high-dimensional PSD feature space. AdaBoost performed poorly overall (accuracy 74.03%, macro F1 62.06%), with a particularly low precision for the anxious class (32.14%), indicating a high rate of false positives. This suggests that AdaBoost struggles with the combination of class imbalance and the nature of the PSD features.

The CNN delivered the best performance across all metrics. It obtained the highest accuracy (95.72%) and macro F1 score (91.88%), which is a 1.37 percentage point improvement over Random Forest. More importantly, the CNN achieved a recall of 83.99% and an F1 score of 86.29% for the anxious class—the best among all classifiers. This highlights the advantage of deep learning architectures for EEG-based anxiety detection, especially when the target class is underrepresented.

These results underscore the CNN’s superiority in handling class imbalance, while highlighting the difficulties that traditional classifiers face even after oversampling is applied to the training set. Importantly, because the test set retains the original imbalanced distribution, the CNN’s superior performance on the minority class demonstrates its robustness to class imbalance under realistic evaluation conditions.

**Figure 3:**
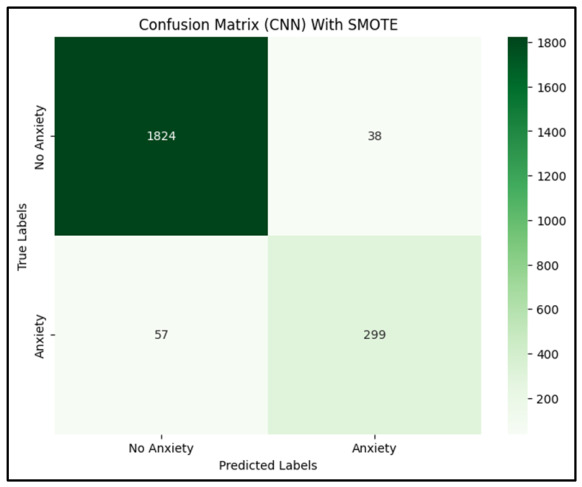
Confusion matrix (CNN).

Figure 3 presents the confusion matrix for the CNN, which demonstrated the best classification performance.

#### 3.7.2. Cross-Validation and Statistical Testing

To further validate the robustness of our findings and to address potential concerns regarding the single hold-out split, we performed 5-fold subject-independent cross-validation on the entire dataset. Table 5 reports the macro-averaged performance across all five folds.

These results are fully consistent with the hold-out evaluation: the CNN again achieved the highest accuracy (95.83%) and macro F1 (91.98%), outperforming RF (accuracy 94.69%, macro F1 89.13%), SVM (accuracy 90.25%, macro F1 82.43%), and AdaBoost (accuracy 74.86%, macro F1 63.17%). The CNN’s superior performance on the minority (anxious) class is also reflected in the aggregated confusion matrix (anxiety recall = 82.55%, macro F1 = 86.43%). These cross-validation results confirm that the CNN’s advantage over traditional classifiers is not an artifact of a particular train/test split, and that it generalizes reliably across different data partitions.

To determine whether the observed differences in cross-validation accuracy (Table 6) are statistically meaningful, we performed non-parametric tests on the per-fold results (5 folds). The Friedman test was used for global comparison across the four classifiers, followed by the Nemenyi post hoc test for pairwise comparisons (α = 0.05). Both tests are appropriate for repeated-measures designs with a small number of folds.

The Friedman test revealed a statistically significant difference among the classifiers (χ^2^(3) = 15.0, *p* = 0.0018). Post hoc Nemenyi tests with adjusted *p*-values indicated that the proposed CNN significantly outperformed AdaBoost (*p* = 0.0456). However, no significant differences were found between the CNN and RF (*p* = 0.92) or between the CNN and SVM (*p* = 0.92). The remaining pairwise comparisons (RF vs. SVM, RF vs. AdaBoost, SVM vs. AdaBoost) were also not significant at the 0.05 level.

These results confirm that the CNN’s advantage over AdaBoost is statistically robust, while its superiority over RF and SVM, although numerically present, does not reach statistical significance with the current 5-fold design.

In summary, the CNN achieved the highest overall accuracy, with notably strong and balanced performance on both non-anxious and anxious states. In contrast, RF, SVM, and AdaBoost showed moderate accuracy at detecting the anxious state, but their precision and recall remained relatively low compared to the CNN.

Beyond performance validation, we also assessed the practical deployability of the CNN by measuring its computational efficiency.

#### 3.7.3. Computational Efficiency

To assess the practical utility of the proposed CNN for real-world anxiety detection, we measured training time, inference time per sample, and model size using the same hold-out validation (the same split as in Section 3.7.1). All experiments were performed on an NVIDIA T4 GPU (Google Colab) using TensorFlow 2.20.0. The CNN was trained for 20 epochs with a batch size of 32 on the preprocessed training set (11,900 samples). Total training time was 204.8 s (≈3.4 min). Inference time was measured on the held-out test set (2218 samples). The average inference time per sample was 0.611 milliseconds, yielding a throughput of approximately 1637 samples/second.

The model contains 14.75 million trainable parameters, which corresponds to a memory footprint of 59 MB when stored as 32-bit floats. This size is modest enough for deployment on modern hardware; for resource-constrained edge devices, the model can be quantized to 8-bit integers, reducing the footprint to ≈15 MB with negligible loss in accuracy. Given that typical EEG segments for anxiety detection range from 1 to 2 s, an inference time of <1 ms means the model can classify each segment faster than real time (i.e., processing a 2 s window in 0.6 ms). This latency comfortably meets the requirements of real-time clinical monitoring systems and wearable EEG headsets.

#### 3.7.4. Most Relevant Features for Anxiety Classification

**Figure 4:**
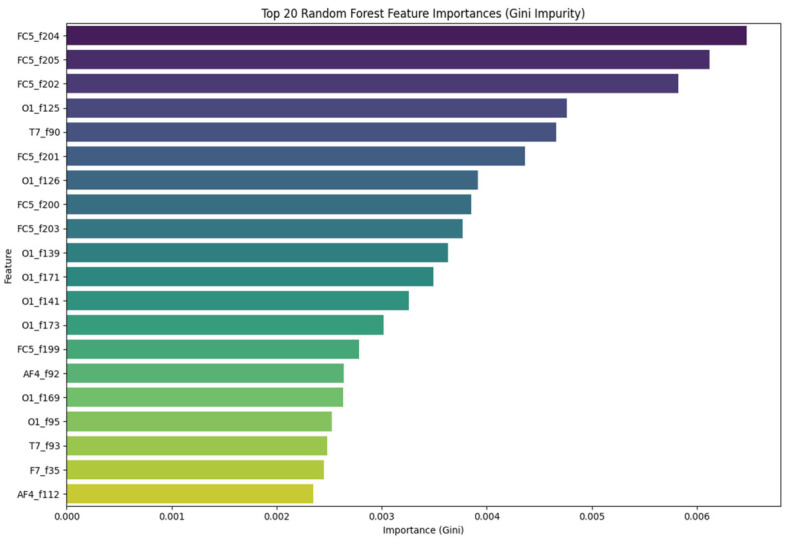
Top 20 most important PSD features for anxiety classification, showing dominant contributions from FC5, O1, T7, AF4, and F7 channels.

The top 20 most important features for anxiety classification, based on RF Gini importance (Figure 4), were all PSD frequency bins. The highest importance came from the FC5 channel (bins f199–f205), followed by O1 (multiple bins), and then T7, AF4, and F7. This indicates that spectral power over frontal (FC5, AF4, F7), occipital (O1), and temporal (T7) regions—especially mid-to-high frequency bins—strongly influenced the model. The prominence of FC5 and O1 suggests that anxiety-related EEG patterns may localize to left frontal and occipital areas, which is consistent with prior neurophysiological research.

#### 3.7.5. Comparison with Related Work

To benchmark our results against existing work, Table 7 compares the performance of the selected classifiers used in this study with other previous studies that employed the same dataset.

In this study, the CNN achieved the highest accuracy of 95.72% for the binary classification task of anxiety vs non-anxiety, indicating its effectiveness at capturing complex patterns in the EEG data. Abdulrahman [36] achieved a maximum accuracy of 90.33% using deep bidirectional long short-term memory (DeepBiLSTM) for multiclass classification. Meanwhile, Lim and Teo [37] reported a classification accuracy of up to 87.89% using regularized class association rules (RCAR). Thus, the performance of the proposed CNN exceeds the best reported results on the same dataset, demonstrating its suitability for classifying emotional states from EEG signals.

## 4. Experiment

This section presents an external, proof-of-concept validation of the trained model on a small, self-collected dataset. The protocol contrasts a stress-inducing Stroop task with a relaxation phase (4–7–8 breathing) to probe state changes in arousal. The experimental protocol comprised two primary components. First, the Stroop test is a custom game environment developed in Unity that was designed to elicit acute cognitive stress and anxiety-related arousal.

**Figure 5:**
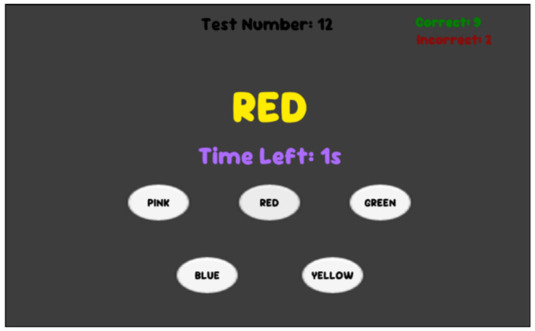
Game interface for the Stroop test.

As shown in Figure 5, it is a standardized measure of cognitive interference where participants identify the color of incongruent stimuli (e.g., “blue” printed in red). The task measures cognitive interference and stress, because participants must focus on the font color rather than the word itself. The Stroop test is widely recognized in EEG research for its ability to induce cognitive conflict and assess emotional responses [4]. Second, the 4–7–8 breathing exercise is a guided relaxation exercise utilized to facilitate emotional recovery and reduce stress levels [41]. Therefore, the experimental protocol should be interpreted as distinguishing acute stress/anxiety-related arousal from relaxation, rather than diagnosing clinical anxiety disorders.

### 4.1. Ethical Statement

This research was approved by the Institutional Review Board (IRB) (project# E-19-3758) of the College of Medicine Research Center (CMRC) at King Saud University (KSU), and the procedures were found to be acceptable on ethical grounds for research involving human subjects. All subjects signed the consent form required by the CMRC-IRB.

### 4.2. Participants

Seven male participants aged between 18 and 60 years were recruited for the experiment, with a median age of 33 years. EEG data were collected by using the Emotiv EPOC X headset, which is a 14-channel wireless EEG device designed for detailed brain data collection.

The headset was calibrated for each participant by adjusting the sensors to fit the participant’s head comfortably, and verifying signal stability to ensure that high-quality EEG signals were acquired. Participants were seated in a quiet and distraction-free room to create an optimal environment for EEG data collection. Any potential interruptions such as noise or visual distractions were minimized. Participants performed stress-inducing tasks and relaxation exercises that encouraged emotional and cognitive reactions.

### 4.3. Assessment Sheet

SAM is a visual questionnaire that uses the Likert scale to measure emotional dimensions such as valence and arousal. In this study, SAM was used to gather additional information about the participants’ emotional state. Each participant completed the SAM twice: once after playing the Stroop test and once after watching the guided exercise video (for the 4–7–8 breathing exercise). A baseline SAM assessment was not collected before the Stroop task; therefore, the SAM ratings were used to compare participants’ self-reported emotional responses after the stress-induction and relaxation phases, rather than changes from a pre-task subjective baseline.

**Figure 6:**
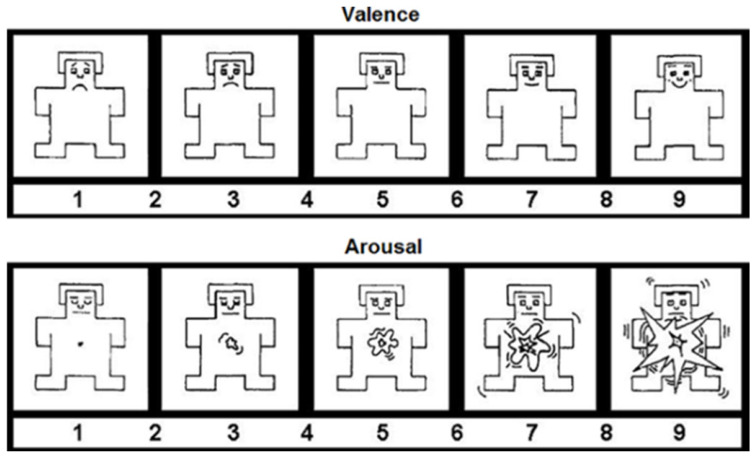
Valence and arousal scales of SAM.

As shown in Figure 6, both the valence and arousal dimensions had scales from 1 to 9. The participants rated their feelings, with 1 representing “very unpleasant” or “very calm” and 9 representing “very pleasant” or “very excited”. The ratings were provided in both English and Arabic to ensure clarity and inclusivity. The pictures simplified the rating process by allowing participants to intuitively and accurately rate their emotional responses [42]. The repeated assessment before and after the relaxation exercise allowed for a comparative analysis of emotional responses.

### 4.4. Experimental Design

**Figure 7:**
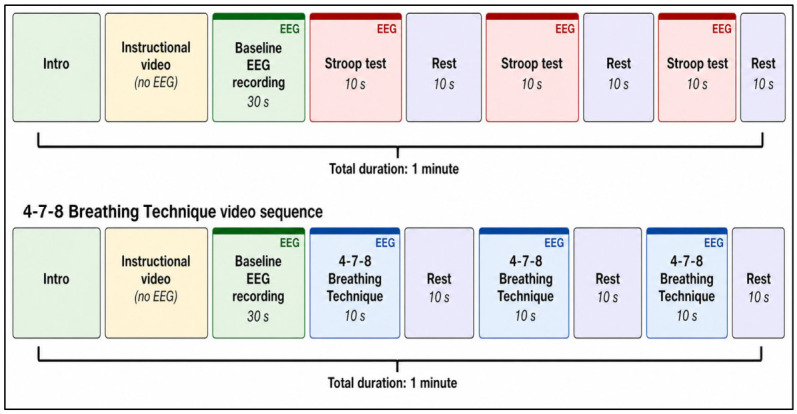
Experimental procedure.

The experimental procedure was designed to systematically collect EEG data and evaluate the emotional responses to cognitive and relaxation tasks. Prior to the experiment, a baseline EEG recording was obtained to capture neural activity in the resting state and to serve as a physiological reference point for quantifying subsequent changes in brain patterns [43]. Figure 7 presents the experimental sequence, which comprised two separate procedures.

For the Stroop test, following the initial baseline recording, participants performed the Stroop test for three consecutive trials. Each trial was separated by a 10 s rest period to prevent cognitive fatigue. EEG data were recorded continuously during the test and rest periods. For the 4–7–8 breathing exercise, following the initial baseline recording, participants viewed an instructional video on the 4–7–8 breathing exercise. The exercise was then performed three times with a 10 s rest interval between repetitions. EEG data were recorded during the video and exercises.

**Figure 8:**
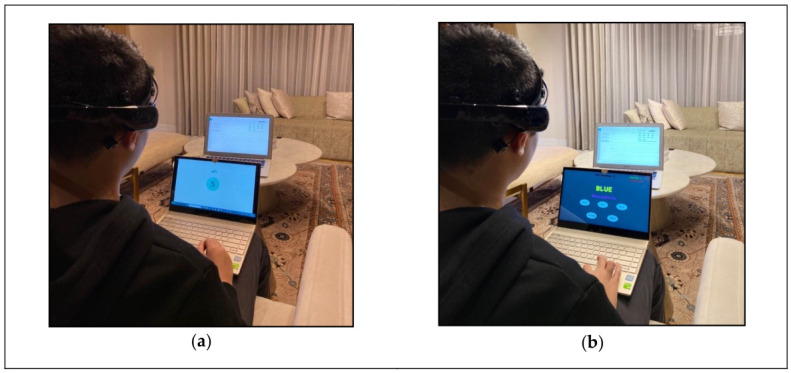
Recording session. (**a**) Participant watching the 4–7–8 breathing technique video. (**b**) Participant playing the Stroop test.

Figure 8 illustrates a participant wearing the headset, performing the Stroop test, and watching the guided exercise video while the EEG waveform is monitored in real time. To prevent visual distractions, the EEG acquisition laptop was placed behind the participant, outside their field of view. The laptop is visible in Figure 8 solely for illustrative purposes; during the actual experiment, participants saw only the stimulus display screen.

### 4.5. Data Analysis

A structured pipeline was utilized to process EEG recordings for training and evaluating a CNN for the binary classification of anxious versus non-anxious states:

1. Preprocessing and Standardization: Raw EEG signals were subjected to a bandpass filter (1–45 Hz) to remove noise while preserving frequency bands relevant to emotional processing [8]. Subsequently, we applied standardization to achieve uniform feature scaling across all channels using the StandardScaler function from the sklearn preprocessing module in the scikit-learn 1.6.1 library.
2. Encoding and labeling: Emotional labels were binarized (Anxious: 1, Non-anxious: 0) based on predefined valence and arousal thresholds derived from the SAM scores (valence < 5 and arousal > 5).
3. Feature Extraction: The continuous signals were segmented using a 4 s sliding window (512 samples) with a 1 s overlap (128 samples). For each window, we computed PSD using the multitaper method. This segmentation strategy captured essential temporal dynamics and maintained consistency with the parameters used during initial model training. Features were extracted directly from the EEG channels (e.g., AF3, AF4, F3, F4) and reshaped to meet the input requirements of the CNN.
4. Train/Test Split: The dataset was randomly split into 80% training and 20% testing sets. To mitigate class imbalance within the experimental data, Borderline-SMOTE was then applied exclusively to the training set (after feature extraction) to generate synthetic samples for the minority class. The test set remained untouched, retaining the original imbalanced distribution.
5. Transfer Learning and Evaluation: A pretrained CNN was fine-tuned on the experimental dataset using transfer learning to adapt pre-learned representations to domain-specific features [44]. Model performance was rigorously assessed on an unseen test set using a comprehensive suite of metrics, including accuracy, precision, recall, and F1 score, supported by a confusion matrix to analyze generalizability.

## 5. Experimental Results

After being fine-tuned on the experimental dataset, the CNN was applied to classify stress-induced anxious and non-anxious/relaxed states. It achieved an accuracy of 86.58%, demonstrating proof-of-concept transferability to the self-collected dataset. SAM summaries (mean ± SD [median]) across participants were: post-Stroop valence = 4.1 ± 1.0 [median 4.0] and arousal = 6.7 ± 0.8 [median 7.0], whereas post-breathing valence = 6.3 ± 0.9 [median 6.0] and arousal = 3.8 ± 0.7 [median 4.0], indicating higher arousal/lower valence after Stroop and the opposite after the 4–7–8 exercise.

**Figure 9:**
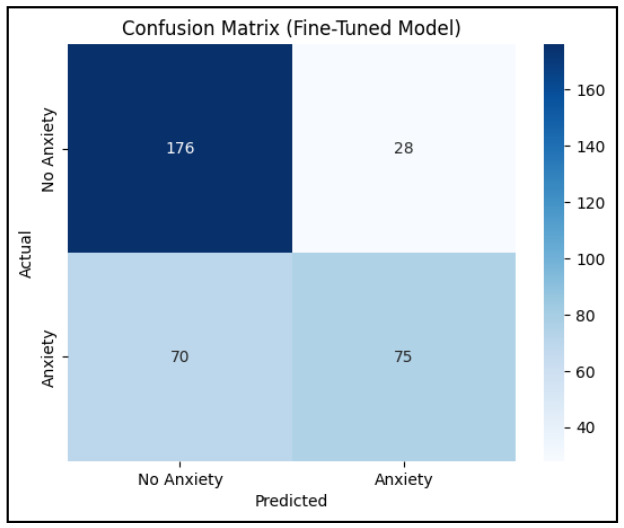
Confusion matrix for the CNN classification performance when applied to the experimental dataset.

These results validate the CNN’s ability to adapt to domain-specific data and highlight its robustness and applicability to classifying emotional states. Figure 9 visualizes the CNN classification performance. The SAM ratings collected during the experiment confirmed consistent variations in self-reported stress/anxiety-related arousal. The Stroop test increased stress/anxiety-related arousal, while the 4–7–8 breathing exercise reduced arousal and supported relaxation.

Given the small number of subjects (*n* = 7), this external validation should be interpreted as a proof-of-concept illustrating the applicability of the trained CNN to new individuals. No claims of population-level generalization are intended or supported. Future work should include a larger, independent, external validation cohort.

## 6. Discussion

The experimental results provide proof-of-concept validation of the proposed system and demonstrate relevance regarding detecting dynamic changes in stress-induced anxious states. The controlled conditions used in the experiment offer a reliable basis for evaluating models for classifying emotional states. However, transfer from GAMEEMO (95.72% accuracy) to our experimental dataset (86.58%) resulted in a 9.14 percentage point decrease. This gap reflects domain shift (recording setups, task paradigms, participant characteristics) and the small experimental sample (*n* = 7). Despite this decrease, the experimental accuracy remains well above chance, suggesting the model captured meaningful EEG differences between stress-induced anxious and relaxed states. Benchmark accuracies should be interpreted as upper bounds; future work should include larger cohorts, cross-subject validation, and domain adaptation.

A further limitation is that the Stroop task primarily induces acute cognitive stress and cognitive load rather than clinical anxiety. Therefore, the experimental findings should be interpreted as classifying stress-induced anxious states versus relaxation/non-anxious states, not as detecting anxiety disorders. We used the Stroop task as an initial proof-of-concept because it is standardized, replicable, and ethically acceptable for inducing cognitive stress and elevated arousal. Future studies should validate the system using clinically validated anxiety-induction paradigms and participants diagnosed with anxiety disorders to assess clinical utility.

Our labeling approach does not capture gradual differences in intensity or mixed states (e.g., high arousal with neutral valence). For example, arousal = 6 and valence = 4 is labeled “anxious”, which plausibly reflects anxiety; arousal = 6 and valence = 5 is labeled “non-anxious”, reflecting a conservative rule that requires clear negative valence. Likewise, arousal = 5 and valence = 4 is “non-anxious” due to insufficient arousal. High arousal with high valence (e.g., 7, 7) remains “non-anxious”, acknowledging that excited states share arousal without negative valence. To address these limitations, future work should consider soft thresholds (e.g., excluding 4–6), treat valence and arousal as continuous targets via regression, or adopt labels from clinical questionnaires rather than self-report only.

Finally, our experimental sample included only male participants due to practical constraints in ensuring stable electrode–scalp contact in our recording setup. Future research should include female participants and individuals with diverse anxiety profiles, including diagnosed anxiety disorders, to better characterize generalizability across populations.

## 7. Conclusions

We developed and evaluated an EEG-based system for detecting stress-induced anxious states using a BCI pipeline with multitaper PSD features and a CNN classifier. On the GAMEEMO benchmark, the CNN achieved 95.72% accuracy, outperforming traditional classifiers. In an external, self-collected dataset (n = 7) contrasting Stroop-induced stress with 4–7–8 breathing, the fine-tuned CNN reached 86.58% accuracy, supporting proof-of-concept transferability. These results indicate that EEG spectral features coupled with deep learning can distinguish stress-related anxious arousal from relaxation.

The current work implements the detection module; integration into a closed-loop neurofeedback system is a direction for future research. Larger and more diverse cohorts, clinically validated induction paradigms, and domain-adaptation strategies are needed to establish clinical utility.

## Figures and Tables

**Table 1:** Related works on EEG-based classification of emotions.

Ref.	Stimuli	Participants	Channels	Selected Channels	Method	Affective States
[8]	Visual-based stimuli	23 (13 female, 10 male)	14	AF3, F7, F3, F4, F8, AF4, FC6, FC5, P7, P8	Emotion classification based on EEG data	Anxiety levels detected
[9]	Mindfulness recording played to adjust mental state	34 (17 with anxiety, 17 healthy)	32	FP1, F3, F7, FP2, F4, F8	Three-stage paradigm: stability, mindfulness, resting state	Anxiety (self-evaluated)
[1]	Exposure therapy (flooding)	23 healthy subjects	14	AF3, F7, F3, FC5, T7, P7, O1, O2	Exposure therapy with flooding and EEG recording	Anxiety levels detected
[11]	Gamified tasks for NF	16 subjects	N/A	N/A	NFB to modulate alpha and high-beta for pain management	Anxiety, depression
[4]	Stroop test, forest scenery (relaxation scenes)	28 healthy adults	32	N/A	EEG classification during VR simulation	Stress, relaxation
[10]	Music videos	32 subjects	5	Fz, AF3, F3, AF4, F4	Emotion classification based on EEG data	Excitement, meditation, boredom, frustration
[12]	Mental imagery tasks (kinesthetic imagination)	30–50 healthy and patient groups	N/A	N/A	User–technology relationship, attention, and spatial abilities	Computer anxiety, fear of incompetence, tension, mood
[13]	IAPS images to induce negative emotions	3 healthy subjects	N/A	FCz-Cpz	Reduce negative emotions using VR and 2D NF by lowering high-beta power	Anxiety, depression, anxiety, emotional regulation
[14]	Video stimuli (positive, neutral, negative)	20 healthy male adults	30	FP1, F3, F7, FP2, F4, F8	NF with real-time feedback and EEG processing	Positive, neutral, negative emotions
[15]	Anxiety-inducing and relaxation scenes with audio	30 young adults (21–35)	4	AF7, AF8, TP9, TP10	BCI-based game with anxiety-inducing and relaxation scenes	Anxiety regulation and attention
[16]	Games (attention and relaxation)	21 young girls	N/A	N/A	Attention and relaxation training via NF games	Anxiety regulation, attention

**Table 2:** Computational techniques used in related work.

Ref.	Dataset	Device Name	Data Preprocessing	Features Extracted	ML Models	Accuracy	Limitations
[8]	DASPS dataset	Emotiv EPOC (14-channel)	Band-pass filter 4–45 Hz, artifacts removed with ICA	PSD and DWT	RF, AdaBoost, SVM, LDA	DWT features + RF + HAM-A labels + SMOTE: accuracy 87.5%	Single DASPS dataset, brief exposure therapy trials
[9]	Collected in the study	Brain Products (32-channel)	ICA and bandpass filter (1–45 Hz)	PSD and frequency-domain power measures using FFT	SVM	92.29% (anxiety subjects), 97.70% (healthy subjects)	Individual differences in cooperation and attitude
[1]	DASPS dataset	Emotiv EPOC	Automatic artifact removal, finite impulse response, bandpass filter	Hjorth features, frequency bands, fractal dimension features	Stacked Sparse Autoencoder	83.50% for two anxiety levels and 74.60% for four anxiety levels	Unbalanced data, subject-independent classification challenges
[11]	Collected in the study	Axon EEG	Filtering	Band power features	N/A	N/A	Small sample size, no control group
[4]	Collected in the study	EMOTIV EPOC	High-pass and low-pass filtering, ICA	Band power features	MLP, SVM	96.42%	N/A
[10]	DEAP dataset	N/A	Bandpass filtering (4.0–45.0 Hz)	PSD, frontal asymmetry, band power	DNN, RF	82% for two classes (valence and arousal)	N/A
[13]	Collected in the study	FlexEEG and VR-HTC Vive Pro 2 headset	Bandpass filter	High-beta band power at midline	Threshold-based	Threshold-based	Very small sample and limited number of sessions
[14]	Collected in the study	NuAmps (Neuroscan)	Bandpass filter (0.1–70 Hz), 50 Hz notch filtering	Frequency-domain power measures obtained using STFT, differential entropy	SVM	Accuracy improved from 56% to 79% w/o video and from 68% to 83% w/video	Male subjects only
[15]	Collected in the study	Muse 2 headband	Bandpass filter	PSD, frequency-domain power measures obtained using STFT	SVM to classify arousal	The SVM model achieved an accuracy of 85% in classifying arousal states	Small sample size, technical issues, lack of long-term data
[16]	Collected in the study	NeuroSky MindWave	N/A	Attention and relaxation levels	N/A	Children kept relaxation and attention indices above threshold for 70% of gameplay	Small sample size, resource limitations

**Table 3:** Description of the GAMEEMO dataset.

**Participants**	28 healthy participants aged 20–27 with no neurological conditions
**Device**	EMOTIV EPOC+ headset (14 channels)
**Channels**	AF3, AF4, F3, F4, F7, F8, FC5, FC6, T7, T8, P7, P8, O1, and O2
**Emotional** **States**	Boring: low arousal, negative valence (LANV) Calm: low arousal, positive valence (LAPV) Horror: high arousal, negative valence (HANV) Funny: high arousal, positive valence (HAPV)
**Experimental** **protocol**	Each participant played four distinct computer games categorized with the emotional labels of boring, calm, horror, and funny. These games were selected based on professional game ratings to target specific parts of the valence–arousal circumplex model. To enhance reliability, participants subjectively rated their experiences post-gameplay using SAM, which were then compared with the emotional categorization based on EEG data.
**Duration**	Participants played each game for 5 min, which resulted in 20 min of gameplay per participant
**Preprocessing**	Down-sampling to 128 Hz to reduce data volume Fifth-order sinc filter to remove high-frequency noise and motion artifacts during gameplay

**Table 4:** Hold-out performance comparison of classifiers in GAMEEMO dataset.

Classifier	Accuracy	Precision	Recall	F1 Score
RF	95.13%	92.78%	88.58%	90.51%
SVM	89.27%	79.65%	83.16%	81.22%
AdaBoost	74.03%	61.14%	66.58%	62.06%
CNN	**95.72%**	**92.85%**	**90.97%**	**91.88%**

**Table 5:** Average cross-validation performance (macro-averaged) of classifiers in GAMEEMO.

Classifier	Accuracy	Precision	Recall	F1 Score
RF	94.69%	94.10%	85.56%	89.13%
SVM	90.25%	81.58%	83.37%	82.43%
AdaBoost	74.86%	62.08%	67.89%	63.17%
CNN	95.83%	93.70%	90.46%	91.98%

**Table 6:** Per-fold accuracy for each classifier in GAMEEMO dataset (5-fold cross-validation).

Classifier	Fold 1	Fold 2	Fold 3	Fold 4	Fold 5	Mean ± SD
RF	94.05%	95.04%	94.77%	94.90%	94.68%	94.69 ± 0.34%
SVM	89.68%	90.04%	89.90%	90.48%	91.16%	90.25 ± 0.53%
AdaBoost	74.30%	74.26%	75.38%	72.12%	78.21%	74.86 ± 1.98%
CNN	94.72%	95.76%	94.82%	96.39%	96.62%	95.66 ± 0.80%

**Table 7:** Performance comparison of classifiers applied to the GAMEEMO dataset in the literature.

Ref.	Feature Extraction	Class Labels	Classifier	Accuracy
**[36]**	14 intrinsic mode functions using VMD and EMD, statistical features calculated	Binary (positive vs. negative) and multiclass (happy, relaxed, bored, stressed)	DeepBiLSTM	90.33% (multiclass) 70.89% (binary)
			KNN	82.21% (multiclass)
			SVM	84.56% (multiclass)
			RF	80.78% (multiclass)
**[37]**	PSD, frequency-domain features obtained using FFT, mRMR feature selection	Multiclass (HAPV, HANV, LAPV, LANV)	Regularized class association rules (RCAR)	87.89%
			Repeated incremental pruning to produce error reduction	79.51%
			SVM	87.29%
**[38]**	PSD features, statistical measures, SMOTE	Multiclass (stress, relaxation, neutral)	RNN	87% (arousal) and 83% (valence)
			RF	83% (arousal) and 75% (valence)
**[39]**	Reservoir computing, genetic algorithm tuning	Valence and arousal	SVM	83.63%
**[40]**	Spectral entropy, normalized data	Binary (positive vs. negative)	BiLSTM	76.91%
**This study**	OSW, multitaper PSD features, standardization, Borderline-SMOTE applied to the training set	Binary (anxious vs. non-anxious)	RF	95.13%
			SVM	89.27%
			AdaBoost	74.03%
			CNN	95.72%

## Data Availability

The project code can be found on Google Collab at: https://colab.research.google.com/drive/1aCKfhTosbYnPZmgZFYYJyDxCeREygdRT?usp=sharing (accessed on 3 June 2026), https://colab.research.google.com/drive/1bWge17lZZZBqpqy--YjyLY8GKNiStDup?usp=sharing (accessed on 3 June 2026), https://colab.research.google.com/drive/18uQ_00ynr0mOT1D6NhArwoxO9RwavTNY?usp=sharing (accessed on 3 June 2026).
